# Current Cervical Carcinoma Screening Guidelines

**DOI:** 10.3390/jcm4050918

**Published:** 2015-05-07

**Authors:** Megan J. Schlichte, Jacqueline Guidry

**Affiliations:** 1 Baylor College of Medicine, 1 Baylor Plaza, Houston, TX 77030, USA; 2 Center for Clinical Studies, 6655 Travis St #120, Houston, TX 77030, USA; E-Mail: jguidry@ccstexas.com

**Keywords:** cervical carcinoma, cytology, HPV testing

## Abstract

A formidable threat to the health of women, cervical carcinoma can be prevented in many cases with adequate screening. The current guidelines for cervical carcinoma screening were created as joint recommendations of the American Cancer Society (ACS), the American Society for Colposcopy and Cervical Pathology (ASCCP) and the American Society for Clinical Pathology (ASCP) in 2012, and later accepted and promoted by the American Congress of Obstetricians and Gynecologists (ACOG). The 2012 recommendations underscore the utility of molecular testing as an adjunct to cytology screening for certain women and provide guidance to clinicians based on different risk-benefit considerations for different ages. This manuscript will review screening techniques and current recommendations for cervical cancer screening and human papilloma virus (HPV) testing, as well as possible future screening strategies.

## 1. Introduction

Routine screening of cervical cytology with the Papanicolaou (Pap) smear has been deeply ingrained in clinical practice for the past century. Originating in the 1940s and recognized as the standard of gynecologic care by the 1960s, annual cervical cytology screening has been an integral part of the well-woman exam for decades [1]. Over the last 30 years, routine cervical cytology screening has contributed to a 50% reduction in the incidence of cervical cancer in the United States [2]. Mortality from cervical cancer has also decreased from 5.55 per 100,000 women in 1975 to 2.38 per 100,000 women in 2008 [2].

The development of cervical cancer is strongly associated with previous infection with the human papilloma virus (HPV) and cervical cancer represents 53.4% of the total number of HPV-associated cancers in women [3]. According to Tota *et al.*, high-risk HPV is a necessary but not sufficient condition for the development of almost all cases of cervical cancer [4]. Therefore, cervical cancer prevention is closely tied to the prevention of HPV transmission and recognition of patients who are infected with HPV and therefore at higher risk for developing cervical cancer. Currently, two vaccines against carcinogenic strains of HPV are commercially available for patients.

Data suggest that more than half of cervical cancer cases could be prevented through adequate screening. Fifty percent of women diagnosed with cervical cancer have never undergone cervical cytology testing and another 10% have not received screening in the five years preceding their diagnosis [2]. Furthermore, cervical cancer is very rare among screened women (less than 10 per 100,000 annually) [5].

The current guidelines for cervical carcinoma screening were created as joint recommendations of the American Cancer Society (ACS), the American Society for Colposcopy and Cervical Pathology (ASCCP) and the American Society for Clinical Pathology (ASCP) in 2012, which were accepted and promoted by the American Congress of Obstetricians and Gynecologists (ACOG) later that same year [2,6]. These guidelines mirror those of the U.S. Preventative Task Force, published at the same time as the joint recommendations [7]. Cervical carcinoma screening should begin at 21 years of age, regardless of age of coitarche or vaccination status, with cervical cytology testing exclusively until age 30. For women 30 to 65 years of age, co-testing with cytology and HPV testing every five years is the preferred method of screening, although cytology screening every three years is acceptable. Screening should be discontinued for women over the age of 65 without a history of cervical intraepithelial neoplasia (CIN) grade 2 or higher and who have had adequate negative prior screening results. In contrast, women with a history of CIN2 or higher should be screened for 20 years following the initial post-treatment surveillance period, regardless of whether or not they have had a total hysterectomy. More intensive screening should be practiced in special populations. Additional discussion pertaining to these and other recommendations based on the 2012 consensus guidelines will follow.

Protocols for cervical cancer screening have varied since their inception, yet these changes have not demonstrated an impact on the life expectancy for patients with cervical cancer [8]. The lack of notable difference is likely related to the indolent course of cervical cancer. Even high-grade cervical dysplasias generally require three to seven years before they develop into invasive cervical cancer and only 30% of untreated CIN3 will develop into cancer over 30 years.

Despite its benefits, cervical cancer screening is not infallible, nor is it universally available. Women without access to medical care, without health insurance, or below the poverty line have an increased risk of developing cervical carcinoma [3]. Additionally, one-third of patients who develop cervical cancer have undergone appropriate screening [8]. Although some of these cases of screening failure may be attributed to lack of patient or provider follow-up, laboratory error, clinician error, misclassification of cancers, aggressive cancers with poor outcome, or cancers undetected by conventional screening, the development of a more sensitive screening method would further minimize morbidity and mortality due to cervical cancer. However, there are ethical limitations to performing randomized control trials to evaluate the efficacy of current cervical cancer screening guidelines [5].

As the prevalence of cervical carcinoma decreases, clinicians as well as the expert panels who advise them must thoughtfully consider the risks involved with cancer screening. These unfavorable consequences include unnecessary colposcopies or biopsies for lesions which might regress, healthcare costs, the psychological impact of a diagnosis of HPV, and an unknown impact on reproductive outcomes [2]. Authors of the most recent recommendations utilize the number of colposcopies as a metric for harm in screening. Colposcopy can led to unnecessary treatment of cervical dysplasia, the current standard for which is the loop electrosurgical excision procedure (LEEP) [5].

The association between LEEP and adverse pregnancy outcomes has been a subject of debate for many years. A meta-analysis found no increase in perinatal mortality associated with a history of LEEP [9]. In 2010, representatives from 22 organizations formed a committee on Practice Improvement in Cervical Screening and Management (PICSM) that reviewed available evidence on cytologic abnormalities in adolescents and found that unnecessary treatment of precancerous cervical lesions can lead to cervical stenosis, preterm delivery, and preterm premature rupture of membranes [10]. ACOG guidelines state that the excision or ablation of cervical tissue in young women should be minimized.

This manuscript will review screening techniques and current recommendations for cervical cancer screening and HPV testing, as well as possible future screening strategies.

## 2. Screening Techniques

### 2.1. Papanicolaou Testing

Newer liquid-based or conventional smear methods of cervical cytology collection are acceptable for screening purposes [2,6]. With liquid-based specimen collection, only one sample is required to perform multiple tests (cytology and testing for HPV, gonorrhea, and chlamydial infection [2]. Though liquid-based specimens theoretically facilitate easier interpretation of results, improved filtration of blood and debris, and fewer unsatisfactory outcomes, comparisons of the liquid-based technique to conventional smear technique have not demonstrated improved sensitivity or specificity for the detection of CIN [11].

Cytologic screening, however, is far from perfect. The ATHENA trial of 47,000 women demonstrated that 10% of women who tested positive for HPV 16 and/or 18 had high-grade cervical disease that was undetected by cytology [12]. In addition, while it has been known for decades that an annual Pap test offers no advantage in terms of clinical outcomes relative to a Pap test every two to three years, the 2012 guidelines represent the first occasion on which leading groups of gynecologists have explicitly recommended against yearly Pap testing [8].

Since healthcare providers may view the Pap smear as the motivation for annual patient visits, clinicians may be reticent to adopt the practice of less frequent testing. However, ACOG still recommends annual appointments for a well-woman exam (even in the absence of cervical cancer screening) [2]. It is important to communicate to patients that recent technological advances in healthcare facilitate comparable protection from cervical cancer even with less frequent screening. Notwithstanding, the limitations of Pap cytology have helped shift guidelines away from exclusive use of morphology-based screening to incorporate molecular-based screening for cervical carcinoma.

### 2.2. HPV Testing

Historically, molecular HPV “reflex testing” has been performed to evaluate the need for colposcopy in women with typical squamous cells of undetermined significance (ASC-US) on cytology (*i.e.*, inconclusive evidence of cervical cancer or its precursors). However, the joint recommendations released in 2012 recommend HPV testing in conjunction with routine cytology as well as in reflex testing starting in women ages 30 to 65 [2,6]. A woman in this age group negative for both high-risk HPV DNA and cytology has a lower risk of developing CIN2 or CIN3 in the next four to six years, relative to a woman with negative cytology alone [2]. In addition, co-testing is more sensitive than exclusive cytology testing in the detection of rare adenocarcinomas of the cervix (accounting for only 10% of all cervical cancer cases, the majority of which are of squamous cell origin) [13].

On April 24, 2014, the FDA approved the cobas^®^ HPV Test as the first of its kind for use in place of cytology for primary screening for cervical cancer by detection of high-risk HPV genotypes in women ages 25 and older [14]. The test provides pooled results for high-risk genotypes (HPV-31, 33, 35, 39, 45, 51, 52, 56, 58, 59, 66, and 68) as well as individual results for HPV-16 and HPV-18. HPV-16 has the highest carcinogenic potential and accounts for 55% to 60% of worldwide cases of cervical carcinoma, while HPV-18 is known to cause 10% to 15% of cervical cancer [15]. There is no indication for performing tests to detect the presence of low-risk HPV genotypes, as these have not been shown to play a role in viral carcinogenesis. In accordance with FDA-approved use of the cobas^®^ HPV Test, women who test positive for HPV genotypes 16 and/or 18 should receive colposcopy and all other women testing positive for high-risk HPV genotypes should receive a conventional Pap test for subsequent triaging. According to large cohort studies, the risk of CIN3 is 10% over one to four years among women who test positive for HPV 16, with the same risk over two to five years among those who test positive for HPV 18 [16,17].

The cobas^®^ HPV test was approved as the primary screening method at three-year intervals, and appears to provide cancer protection very similar to co-testing at three-year intervals or annual cytology, and superior protection relative to cytology at three-year intervals [5]. The approval of the cobas^®^ HPV test starting at age 25 (rather than 30, as indicated in the 2012 guidelines) aligns with the ASCCP 2012 updated recommendations for the management of abnormal tests [18]. In response to FDA approval, interim guidelines for primary high-risk HPV screening were developed by representatives from the Society of Gynecologic Oncology, the American Society of Cytopathology, and the College of American Pathologists, in addition to ACOG and all groups authoring the 2012 screening guidelines [19]. The interim guidelines state that because of equivalent or superior effectiveness, primary high-risk HPV screening can be utilized as an alternative to cytology. Primary HPV screening should not be implemented among women younger than 25 years of age and re-screening after a negative primary high-risk HPV test should not take place more frequently than every three years. In addition, although more research is necessary, the interim guidelines suggest that the best management of high-risk HPV-positive women is to triage positive tests with genotyping for 16/18 and to utilize reflex cytology for women positive for the 12 other high-risk genotypes.

There is a growing body of evidence in support of these expert panel interim recommendations. The Joint European Cohort study demonstrated that the six-year risk of CIN3 or higher following a single negative HPV test was significantly lower than the risk following a negative Pap in a group of 24,295 women [20]. An observational study conducted in Portland suggested that a single HPV test could predict risk of CIN3 or higher for 18 subsequent years [21]. Moreover, a landmark observational study [22] as well as a systematic review [23] suggested that co-testing may provide only marginal benefit when compared to exclusive HPV testing.

As additional tests are approved by the FDA for primary screening of cervical cancer, the choice of HPV assay may present challenges. A 2014 study of 5064 SurePath samples of women participating in routine cervical screening found considerable disagreement between four human papillomavirus assays, Hybrid Capture 2, cobas, CLART, and APTIMA [24]. The extent of discordance between primary screening results of the assays was neither population- nor storage media-specific, suggesting that assay design differences were the most likely cause [24]. These findings emphasize the need for quality control of HPV assays and special attention to the follow-up of women with HPV positive and cytology normal results.

Nevertheless, multiple studies provide mounting evidence for the utility of HPV testing as primary screening and may continue to lead to further changes in recommendations, particularly as the actual impact of new screening guidelines on cancer prevention is investigated [25]. Furthermore, in countries with HPV vaccination programs, HPV testing may also offer a cost-effective strategy to monitor long-term vaccine efficacy [4]. For clinicians who recognize the value of HPV testing, but who are reticent to adopt less frequent cytology screening in accordance with the 2012 consensus guidelines, it is possible that HPV testing will increase in the absence of a concomitant decrease in the frequency of cytology screening, thus driving up healthcare costs. In addition, frequent screening will increase the number of false positives and unnecessary procedures, with increased potential harm to the patient.

## 3. Screening Guidelines

### 3.1. Level A Recommendations, Based on Good and Consistent Scientific Evidence

#### 3.1.1. Initiation of Screening

According to unanimous agreement of ACOG, ACS, ASCCP, ASCP, and USPSTF, cervical carcinoma screening should begin at 21 years of age. Young women are commonly infected with HPV within just a few months to a few years after coitarche, but the infection is nearly always cleared by a young, immunocompetent person within one to two years [26]. Women under 21 have a very low incidence of cancer (0.1 percent cases diagnosed before age 20) and there is a lack of evidence to suggest that screening effectively reduces the rate of cervical cancer in the under-21 demographic [2,6]. This recommendation is independent of sexual history or behavioral risk factors. Additionally, non-malignant cervical neoplasia is not uncommon in the under-21 age group and abnormal Pap tests may promote unnecessary anxiety, morbidity, and follow-up workups and procedures. In spite of the recommendation against cervical carcinoma screening in this age group, it should be noted that women younger than 21 should still be counseled about safe sexual practices and receive vaccination against HPV as part of their primary care.

#### 3.1.2. Screening for Women Ages 21 to 29

Women between the ages of 21 and 29 should receive cervical cytology screening alone every three years (rather than every two years, as previously recommended) [2,6]. Co-testing to detect the presence of high-risk HPV genotypes should not be performed in women under 30 years of age. While HPV testing is more sensitive than cervical cytology testing, it is less specific. Thus, given the high prevalence of high-risk HPV infections in women less than 30 combined with their lower risk for cervical cancer due to probable immune system eradication of transient HPV infection, screening by co-testing is not recommended. Co-testing in this age group would lead to an increase in unnecessary workup procedures without a corresponding decrease in cervical carcinoma incidence [2].

The evidence for the increase in the interval of screening stems from modeling studies that found no significant difference in the predicted lifetime risk of cervical cancer in women screened every three years (five to seven new cases per 1000) *versus* every two years (four to six cases per 1000) [26]. Furthermore, under this model, women screened every two years would undergo 40% more colposcopies than women screened every three years [27].

#### 3.1.3. Screening for Women Ages 30 to 65

Women between 30 and 65 years of age should be co-tested with cytology and HPV testing every five years (instead of every three years, as previously recommended) [2,6]. Alternatively, this age group can be tested with cytology alone every three years. According to ACOG, ACS, ASCCP, and ASCP, co-testing is preferable to conventional cytology testing alone because of the increased sensitivity in detection of CIN3 and cervical cancer; however the USPSTF recommends either mode of screening without preference [6,7]. With the increased interval of screening every five years with co-testing, specificity is maintained relative to cytology alone every three years [2,6]. Approximately 10% of cytology results return as ASC-US in women 30 to 65 years of age and most of these cases should receive reflex HPV testing [8]. However, a shift in practice to adopt the new screening guidelines will promote HPV testing in a much greater percentage of women in this demographic as part of routine co-testing.

Justification for lengthened screening intervals is based on the principle of equal management for equal risk. Because multiple possible screening results are associated with similar risks of the presence of cervical cancer precursors or cancer, as well as the risk of colposopies and other procedures, patients should be managed consistently based on that risk [5]. Data suggests the risk of CIN3 or higher five years after a negative co-test is equal to or less than the risk three years after a negative Pap [28]. However, the five-year co-test protocol has yet to be universally accepted due to patient and clinician concerns about inadequate protection from cervical carcinoma under the new guidelines. Kulasingam *et al.*’s model suggests that implementation of five-year co-testing will lead to one additional cervical cancer diagnosis out of 369 appropriately-screened women and one additional cervical cancer death among 1639 screened women, as compared to three-year co-testing [28].

According to data from Kaiser Permanente Northern California (KPNC) based on screening results from 1,008,855 women, 2734 additional cervical cancer diagnoses and 615 deaths from cervical cancer would be prevented if the three-year co-testing interval is maintained in lieu of the five-year interval. If instead a two-year co-testing interval was adopted, each cancer prevented would require 92 additional colposcopies and each death prevented would require 408 additional colposcopies [5]. While the additional risk inherent in the longer screening interval may be small in the short-term, the concerns of patients and clinicians about the current interval recommendation is reasonable in light of a lifetime, cumulative risk of cervical cancer [5].

#### 3.1.4. Discontinuation of Screening

According to the latest consensus guidelines, all screening should be discontinued for women after the age of 65 who have had adequate negative screening [2,6]. Adequate negative screening is defined as three consecutive negative Pap tests or two consecutive negative co-test results within 10 years, with the most recent test performed within the past five years. This recommendation only applies to those women older than 65 who do not have a history of CIN2 or higher within the last 20 years and who do not have increased risk of cervical cancer due to other exposures. The USPSTF has already adopted this recommendation.

Even if a woman older than 65 has a new partner, the low risk of HPV persistence and progression to cancer with newly acquired HPV infection renders any screening in women over 65 with adequate negative prior screening results unnecessary. Post-menopausal epithelial atrophy, common in this age group, can lead to false-positive results and thus abnormal cervical cytology has a very low positive predictive value [2]. Furthermore, smaller and less accessible cervical transformation zones found among many post-menopausal women could require more interventions to provide adequate tissue specimens and ultimately treat patients [13]. The slight benefits in terms of cervical cancer diagnosis and mortality are outweighed by significant overall cost from unnecessary screening, anxiety, and colposcopy procedures. Based on modeling studies, continuation of screening until age 90 prevents only 1.6 cancer cases and 0.5 cancer deaths and extends life expectancy by only one year per 1000 women screened [28].

The ACS, ASCCP, and ASCP did not find sufficient evidence to suggest that the frequency of screening in any age group should be altered based on prior negative cytology [6]. A matched case control study demonstrated that neither a previous abnormal cytology result nor the number of previous normal screening results changes the risk of invasive cancer [29].

#### 3.1.5. Screening of Special Populations

For women with a history of cervical cancer, previously treated CIN2, CIN3, existing HPV or HIV infection, immunodeficiency, or a history of diethylstilbestrol exposure in utero, recommendations for screening are more intensive. According to the CDC, HIV-infected women should be screened by cervical cytology twice in the first year following diagnosis and yearly thereafter, regardless of whether or not the diagnosis of HIV is made prior to the age of 21 years [30]. The consensus conference review of the ACS, ASCCP, and ASCP specifically states that it does not address patients who are HIV+, who should follow the CDC guideline [6]. By strict interpretation, the CDC recommendation would suggest that female children or even infants who are positive for HPV by vertical transmission be screened; thus, clinical judgment should be used. For patients after solid organ transplants or with other causes of immunosuppression, ACOG suggests that annual cytology is reasonable. No explicit recommendations have been made for HPV testing among those with immunodeficiency due to HIV or other factors.

#### 3.1.6. Screening for Women after Hysterectomy for Benign Reasons

Among women who have had a total hysterectomy, that is, inclusive of removal of the cervix, and who have never had CIN2 or higher, routine cytology screening and HPV testing should be completely discontinued [2,6]. Continued cytology testing in this population is ineffective, given the extremely low risk and rarity of vaginal cancer. Evidence that supports this recommendation was noted in a systematic review of 19 studies involving a total of 6543 women who had a hysterectomy without a history of CIN and 5037 women who had a hysterectomy in which the cervix was affected by CIN3 [31]. Among those who had a hysterectomy in the absence of history of CIN, 1.8 percent had an abnormal cytology screening result, and 0.12 percent had vaginal intraepithelial neoplasia on biopsy, with no reported cases of cancer. 

### 3.2. Level B Recommendations, Based on Limited and Inconsistent Scientific Evidence

#### 3.2.1. Follow-Up Screening for Women with ASC-US Cytology or Discordant Co-Testing Results

Consistent with the 2013 ASCCP updated guidelines for the management of abnormal screening results [18], the ACS recommended in their 2015 screening update that women with ASC-US cytology and negative HPV co-testing should return for screening in three years [32]. This three-year screening interval, reduced from five years, as previously recommended in the 2012 consensus guidelines, is based on newer evidence from the KPNC database to include 1.1 million women and two additional years of follow-up, which found that the risk of pre-cancer or cancer after the ASC-US/HPV-negative result was not as low as that associated with a negative co-test [32,33]. Instead, the risk approximated that of a negative Pap test (with no HPV result), for which repeat screening is recommended after three years [33]. It should be noted that the overall risk of malignancy in this population is still low, 0.5% or less over five years, rendering colposcopy a poor diagnostic option [32].

In contrast, if the HPV test is positive in the context of negative cytology, clinicians may either repeat co-testing in one year or perform an HPV genotyping test. Immediate colposcopy is indicated in cases of either subsequent detection of high-risk HPV genotypes (16 or 16/18) or a cytology result of LSIL or higher with a repeat positive HPV test upon one-year follow-up [2]. For cases in which HPV-16 or HPV-18 is detected, the risk of CIN3 approaches 10% within a few years [12]. However, cohort testing by Rodriguez *et al.*, suggests that many HPV infections will be cleared by the immune system within one year [34].

#### 3.2.2. Screening for Women with a History of Cervical Dysplasia or Cancer, with or without Hysterectomy

For women previously treated for CIN2 or higher, there is a 2.8-fold increased risk of persistent or recurrent disease for 20 years following treatment that necessitates continuation of routine age-based screening [2,6]. This recommendation applies even if the 20-year post-treatment screening period extends past the age of 65 years in women who have had a history of CIN2 or higher. While women older than 65 represent 14.1% of the U.S. population, they account for 19.5% of new cases of cervical cancer, usually due to inadequate screening [2]. Recurrent intraepithelial neoplasia or carcinoma can develop at the vaginal cuff years after hysterectomy. No explicit recommendations have been made for or against HPV testing in this demographic.

### 3.3. Level C Recommendation, Based on Consensus and Expert Opinion

#### Screening and the HPV Vaccination

Regardless of HPV vaccination status, the current recommendation is to screen women according to standard age-based guidelines [2,6]. While the Advisory Committee on Immunization Practices and CDC recommend that women receive vaccination against HPV-16 and HPV-18 prior to the time of HPV exposure. However, many women are currently vaccinated subsequent to HPV exposure [2]. Thus, clinicians may not observe a marked reduction in cervical cancer incidence for 20 years following widespread implementation of vaccination [2]. First introduced in 2006, vaccination against HPV has been shown to protect previously uninfected women against CIN caused by HPV-16 and HPV-18 in nearly all cases [2].

While additional studies are still necessary, it is reasonable to suggest that among individuals vaccinated before the initiation of sexual activity, future guidelines may permit screening in women beginning at age 25 [5]. However, vaccinated individuals remain unprotected from 30 percent of cervical cancer cases due to other HPV genotypes not included in the vaccine [35]. Vaccination compliance rates are also poor, as evidenced by CDC data documenting that only 36.9% of women aged 19 to 26 years reported receiving more than one dose of the HPV vaccine in 2013 [36]. In light of these realities, at the present time the best prevention measure against cervical carcinoma is widespread screening for women independent of HPV vaccination status.

## 4. Future Screening Strategies

Screening guidelines will continue to evolve with increasing understanding of the relationship between HPV oncoproteins and cervical dysplasia. While highly sensitive, current HPV testing methods lack specificity and positive predictive value [13]. Women with a negative Pap test who test positive for high-risk HPV may have a risk of only 3% to 7% for high-grade CIN, but a positive HPV test could prompt unnecessary diagnostic workup and treatment [37,38]. The biomarkers p16, Ki-67, human telomerase reverse transcriptase (hTERT), and L1 capsid protein hold clinical promise for more accurate diagnostic workup of patients with positive HPV testing. While p16 is overexpressed in cervical dysplasia and associated with high-risk HPV oncogenic transformation, Ki-67 and hTERT are markers of uncontrolled cell proliferation [13,39]. Meanwhile, the presence of HPV L1 capsid protein within dysplastic cells demonstrates a completed HPV life cycle, and HPV L1 capsid protein-negative cases have lost the capacity to synthesize virions [40]. The combination of mRNA expression levels of several of these HPV biomarkers could be used in a complementary manner in diagnosing high-grade cervical lesions [41]. For example, a 2011 study found that dual-stained cytology, a combined assay for both the p16 and Ki-67 markers, was 91.9% sensitive and 82.1% specific in detecting CIN2 or higher and 96.4% sensitive and 76.9% specific in detecting CIN3 or higher [41]. Application of dual-stained cytology may be particularly useful in helping to triage patients with ASC-US cytology and/or in cases of women with a positive HPV test and negative Pap test.

Because HPV testing is more sensitive than cytology, although less specific, a reasonable future screening strategy may be testing for HPV first followed by a conventional Pap test in women with a positive HPV test result. The multi-stage HPV FOCAL trial of 6154 women compared HPV followed by Pap testing *versus* Pap followed by HPV testing (with the latter group comprised of women with ASC-US on cytology) [42]. The first round of results demonstrated increased detection of CIN2 or higher by HPV DNA testing followed by cytology relative to the reverse order of triage. This screening strategy, in combination with testing for the new biomarkers mentioned previously, holds much promise for providing a screening methodology that provides the most significant protection from cervical cancer.

## 5. Conclusions

As molecular screening and vaccination slowly decrease the need for traditional cytological testing, clinical practice guidelines will continue to change. Significant research advances have been made in the prevention, screening, and treatment of cervical carcinoma and will likely continue. HPV testing received a strong endorsement in the 2012 consensus guidelines, yet has not been fully accepted by healthcare providers. A recent survey of 266 obstetricians and gynecologists demonstrated that compliance with guidelines is suboptimal, with solo practitioners especially unlikely to follow both vaccination and screening guidelines relative to those in group practices [43].

In the survey, many physicians also cited patient and personal discomfort with extended screening intervals. Therefore, provider and patient education about the benefits of lengthened screening intervals (conservation of healthcare resources, lack of increased risk) in women who are at low-risk for cervical carcinoma is needed [44]. To minimize unnecessary surveillance and invasive workups for women with a low risk of cervical cancer, evidence for the use of novel biomarkers better able to detect high-grade dysplasias should be carefully considered. In particular, tests for p16 and Ki-67 as well as HPV mRNA assays hold much promise.

Despite the improvements in screening technology, women without access to adequate health care will not benefit from these advances. Targeted HPV vaccination efforts in lower socioeconomic populations may be the key to reducing high rates of cervical cancer incidence and mortality [45]. The incidence of cervical cancer in areas where more than 20% of U.S. residents live under the poverty line is 18% to 39% higher than areas where less than 10% of residents live below the poverty line [46]. Greater sexual activity and lower screening participation in these populations may synergistically promote higher cervical cancer risk, and vaccination of these high-risk groups may reduce disease burden [45]. In a climate of changing recommendations, as well as new tests, vaccines, and treatments, both quality research investigations and education of clinicians remain essential.
